# Harmonize: a shared environment for extended immersive entertainment

**DOI:** 10.1007/s10055-021-00585-4

**Published:** 2021-10-07

**Authors:** Damiano Oriti, Federico Manuri, Francesco De Pace, Andrea Sanna

**Affiliations:** Dipartimento di Automatica e Informatica, Politecnico di Torino, Corso Duca degli Abruzzi, 24, 10129 Torino, Italy

**Keywords:** Augmented reality, Collaborative environments, Immersive entertainment, Immersive environments, Shared environments, Virtual reality

## Abstract

Virtual reality (VR) and augmented reality (AR) applications are very diffuse nowadays. Moreover, recent technology innovations led to the diffusion of commercial head-mounted displays for immersive VR: users can enjoy entertainment activities that fill their visual fields, experiencing the sensation of physical presence in these virtual immersive environments. Even if AR and VR are mostly used separately, they can be effectively combined to provide a multi-user shared environment (SE), where two or more users perform some specific tasks in a cooperative or competitive way, providing a wider set of interactions and use cases compared to immersive VR alone. However, due to the differences between the two technologies, it is difficult to develop SEs offering a similar experience for both AR and VR users. This paper presents Harmonize, a novel framework to deploy applications based on SEs with a comparable experience for both AR and VR users. Moreover, the framework is hardware-independent, and it has been designed to be as much extendable to novel hardware as possible. An immersive game has been designed to test and to evaluate the validity of the proposed framework. The assessment of the system through the System Usability Scale questionnaire and the Game Experience Questionnaire shows a positive evaluation.

## Introduction

Augmented reality (AR) and virtual reality (VR) applications are very diffuse nowadays due to their ability to enhance and to simplify human tasks in many fields. Milgram and Kishino first defined AR, VR and their relation in the *reality–virtuality continuum* in 1994 (Milgram et al. 1994). A VR environment is one in which participants are immersed in a synthetic world, which can be either realistic or fantastic. AR environments consist of virtual objects anchored to specific positions in the real world, and they are aimed at “augmenting natural feedback to the operator with simulated cues” (Milgram et al. 1994).

Immersive environments (IEs) are simulations that fill the user’s visual field, giving the sensation of physical presence (Getchell et al. 2011; Rubio-Tamayo et al. 2017). The term immersive entertainment defines all the entertainment activities which take place in a IE. Cave Automatic Virtual Environments (CAVEs) are the oldest example of immersive virtual environments and consist of a cube-shaped room where projectors are directed towards 3 (up to 6) walls (Cruz-Neira et al. 1992). VR environments allowing the presence of more than one user at the same time for collaboration are called collaborative virtual environments or shared virtual environments (SEs).

VR applications are traditionally experienced with computer monitors; thus, users can get distracted by real-world stimuli and it could be difficult for them to feel a real sense of *presence* (North and North 2016). However, recent technology innovations led to commercial head-mounted displays (HMDs) for VR, such as the Oculus HMDs^1^ or the HTC Vive.^2^The videogame industry is greatly investing in immersive entertainment, with the gaming market size valued at USD 11.56 billion in 2019 and expected to grow at a compound annual growth rate of 30.2% from 2020 to 2027 (GrandViewResearch 2020). Immersive entertainment can greatly benefit from SEs, by connecting people who live apart and making them feel close. The ability of sharing immersive virtual worlds with people far away connected through the Internet can be a way of circumventing the movement restrictions and isolation caused by the recent Covid-19 pandemic. Moreover, even if AR and VR are mostly used separately, they can be effectively combined together in a SE, providing a wider set of interactions and use cases. For example, a museum guide may provide a tour in a real museum and see remote visitors through AR, whereas the latter enjoy the artworks in high resolution through a detailed, immersive VR reconstruction of the site. Performers and artists may work from their home, perceiving the presence of the audience through AR, whereas the public experiences the show through immersive VR.

This paper presents Harmonize, a novel framework to deploy applications based on SEs for AR and VR users. Harmonize was built on top of Unity3D engine, in order to take advantage of a free^3^ and powerful engine offering state-of-the-art authoring tools, e.g. for animation and scene creation. In order to test and to evaluate the validity of Harmonize, an immersive game has been developed to offer a similar gameplay experience to both AR and VR users.

The proposed framework has been developed and tested with the Oculus Rift CV1 VR HMD and the Microsoft HoloLens AR HMD. Although the Oculus device is relatively affordable, the Microsoft product is rather expensive, being oriented to business customers and researchers. The choice of using the Microsoft HoloLens is justified by two factors: 1) its tracking accuracy is comparable to the accuracy of the Oculus Rift CV1 tracking system, 2) it does not constraint the user hands as handheld AR solutions do. The price of see-through AR HMDs should significantly decrease in the future, thus making the proposed approach commercially viable. On the other hand, both AR and VR clients could be implemented by a cardboard and a smartphone; this solution would penalize the AR client as the real world is mediated by the device camera. Another factor that affects the cost of a AR/VR framework is the virtual reconstruction of environments, which may require many hours of work of a skilled 3D artist or the usage of expensive solutions like LiDAR. Although the presented work is based on manual reconstruction of the real environment, an extension of this framework using automatic deep learning-based 3D reconstruction is being worked on.

The major contributions of the presented work are the following: (1) a complete framework to develop multiuser applications for AR/VR, (2) an integrated networking system designed for fast paced games, (3) a system based on spatial anchors to align a virtual scene to its real counterpart, and (4) a study of a multiplayer game where both AR and VR players share the same environment and play with the same rules and modalities.

In the next section, the major problems in developing frameworks for multi-user AR and VR applications and the most relevant works known in the literature are reviewed. The architecture of the proposed framework and its implementation are presented in Sects. 3 and 4. Section 5 provides an overview of a generic application developed with Harmonize. Section 6 describes the immersive game developed to test the framework, whereas Sect. 7 describes the tests performed and an analysis of the obtained results.

## Previous works

In order to develop a multi-user application tailored for both AR and VR users, developers have to usually deal with the specific libraries and software development kits (SDKs) of the target devices they want to support. In fact, while with mice and keyboards it is not necessary to program for a specific brand of device, in order to support AR and VR devices it is common to have to deal with proprietary SDKs. Moreover, when applications have some kind of sharing feature using the network infrastructure, it might be necessary to cope with network libraries as well. Finally, in order to support full distributed immersive worlds, developers have to address some general problems that were identified by Broll (1997): (1) keeping shared worlds consistent, (2) the network protocol must scale to (large) number of users, (3) consideration of reliability issues versus interactivity, (4) support of cooperation rather than coexistence, (5) heterogeneous network connections and 6) composition of large-scaled subdivided worlds.

DIVERSE is a modular VR framework proposed by Kelso et al. (2002) and based on existing software packages. Although it was designed to facilitate the creation of device-independent virtual environments, it was not designed with AR in mind.

VHD++ (Ponder et al. 2003), MORGAN (Ohlenburg et al. 2004) and Instantreality (Behr and Fellner 2011) are modular and extensible frameworks for AR and VR. VHD++ comprises a runtime engine, some plugable components called services, and a hierarchy of data objects for both system and simulation states. MORGAN presents a component-based architecture and uses well-known software patterns such as the *publisher-subscriber* pattern for managing different devices, and the *factory method* pattern for component creation and instantiation. It also supports SEs and concurrent manipulation of the scene by multiple users. Instantreality, designed for industrial applications, achieves tracking through marker-less computer vision techniques such as feature tracking and inertial sensors. Although VHD++, MORGAN and Instantreality are quite powerful frameworks, they are difficult to maintain due to their complexity and they do not provide integrated and easy to use tools for scene creation and management.

Developed by Anthes and Volkert (2006), inVRs is a framework for networked VR applications written in C++ and offering a modular design, allowing its different modules to be used as separate libraries. CalVR (Schulze et al. 2013) is an open-source VR middleware based on OpenSceneGraph^4^ and offering collaborative session support. Thanks to its modular architecture, CalVR allows compiling new modules separately from the main code using a plugin system. CocoVerse (Scott et al. 2017) is a shared immersive environment in which users can interact with each other and collaborate to create virtual objects using a set of predefined tools. The application employs the HTC Vive and related motion controllers, making it possible to track the user motion in a room-scale volume. CalVR, inVRs and CocoVerse were designed for VR; thus, they are not tailored towards SEs mixing AR and VR users.

ARTiFICe (Mossel et al. 2012) is one of the most recent frameworks. Since ARTiFICe is based on the Unity3D engine, it leverages its multiplatform support, its native support of common device types and the integrated authoring tools for scene creation. To the best of the authors knowledge, ARTiFICe does not support modern devices such as HoloLens and it does not offer a uniform experience regardless of the hardware of choice.

VREX (Blonna et al. 2018) is a VR game framework based on Unity3D and the HTC Vive, a popular VR HMD, providing tools for managing the player interactions, “creature” creation and movement, and game objectives. VREX does not support AR devices.

Casarin et al. (2018) propose an implementation of UMI3D (Casarin et al. 2017), a unified model for interaction in 3D environment, based on the popular Unity3D engine. They introduce a toolbox that helps to develop AR or VR applications designed for cooperative work. The proposed tools allow the developers to manage interactions, synchronisation and graphics, but they lack embodiments (i.e. avatars).

ARCalVR (Zhang et al. 2019) is an extension of CalVR aimed at supporting Android smartphones as AR devices. The main additions to the original VR framework are new tracking and environment understanding features and an improved user-interaction interface, which has been adapted in order to support finger controls in place of mouse clicks for 3D dragging and rotations.

Bozzelli et al. (2019) design an integrated AR/VR framework tailored for user-centric experiences of cultural heritage. The framework is used to develop two different applications as part of a project called ArkaeVision, one for AR (ArkaeVision Art) and one for VR (ArkaeVision Archeo). The developed experiences have different goals, visualisations and interaction modalities.

Albeit the described frameworks offer several functionalities and support extensibility, even the most recent ones present two relevant problems: firstly, most of them are not designed to support a shared environment with both AR and VR users at the same time; secondly, even if some of them could be theoretically modified to provide this support, they are not designed to offer a comparable experience regardless of the hardware of choice, either AR or VR. Thus, a framework supporting modern AR and VR devices and aimed at creating SEs that could be experienced evenly with both AR and VR devices at the same time is still missing. The purpose of this research is to develop a novel framework, *Harmonize*, to bridge that gap. Moreover, it has been designed to facilitate the creation of shared immersive experiences for entertainment, removing the burden of managing common low level tasks related to the application lifetime from application developers.

## Architecture and design choices

**Fig. 1:**
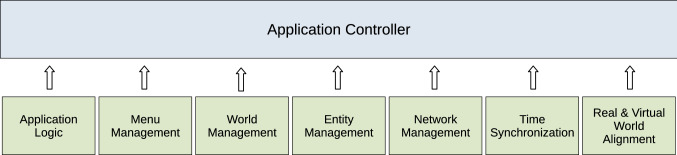
The framework architecture with application controller and modules. The application logic refers to the application mode, which is defined by a set of rules. The network management module handles connections and network messages. The real and virtual world alignment module is needed to support AR systems

After an analysis of the existing frameworks and applications, it was possible to define the requirements for the proposed framework. First, developers should be able to easily add both the rules defining the scope of the intended application, and new entity classes. Moreover, the framework should handle autonomously all the other factors of a networked application, such as connections, distributed computation and synchronisation. In order to develop and test the overall system more efficiently and effectively, the framework was designed in a modular fashion (see Fig. 1). For every major functionality, such as networking and entity management, an independent module has been developed and all the modules are administrated and coordinated by an application controller.

### User input management

Modern AR and VR SDKs and frameworks assure the forward-compatibility of the application in terms of input, which means that applications developed with these toolkits will be compatible with future devices. This is obtained virtualising raw inputs in terms of application logic, which is based on actions that can be triggered by inputs. When a new device comes out on the market and the toolkit is updated, the upgraded runtime will provide the proper input mapping, thus avoiding extensive code-rewriting to make the application compliant with the new device. The idea of abstracting actions from raw inputs has been adopted by the Mixed Reality Toolkit (MRTK), the SteamVR 2 SDK and OpenXR, the open standard by the Khronos group. Thus, Harmonize takes advantage of the decoupling of the application logic from the actual hardware to guarantee compatibility with future devices. Moreover, the proposed framework has been designed to automatically detect the user device type and to allow users to choose the preferred interaction paradigm among those supported by the selected hardware.

### The shared world structure

Some requirements have to be satisfied in order to guarantee an even experience when AR and VR users share the same environment. One of such requirements dictates that the virtual environment should be constructed as topologically similar to the real play spaces as possible. Two design choices have been considered: (1) reconstructing on the fly a 3D representation of the actual location of the AR users and then sending the 3D reconstruction to VR users; (2) modelling, beforehand, a specific location with traditional 3D modelling techniques and then including it in the application asset database. Reconstruction methods such as KinectFusion (Izadi et al. 2011) are very computationally intensive; thus, they are too demanding for current AR hardware; moreover, the reconstruction quality is not detailed enough to be satisfying for a VR experience. For these reasons, the current version of Harmonize supports traditionally generated environments.

### The VR locomotion method

**Fig. 2:**
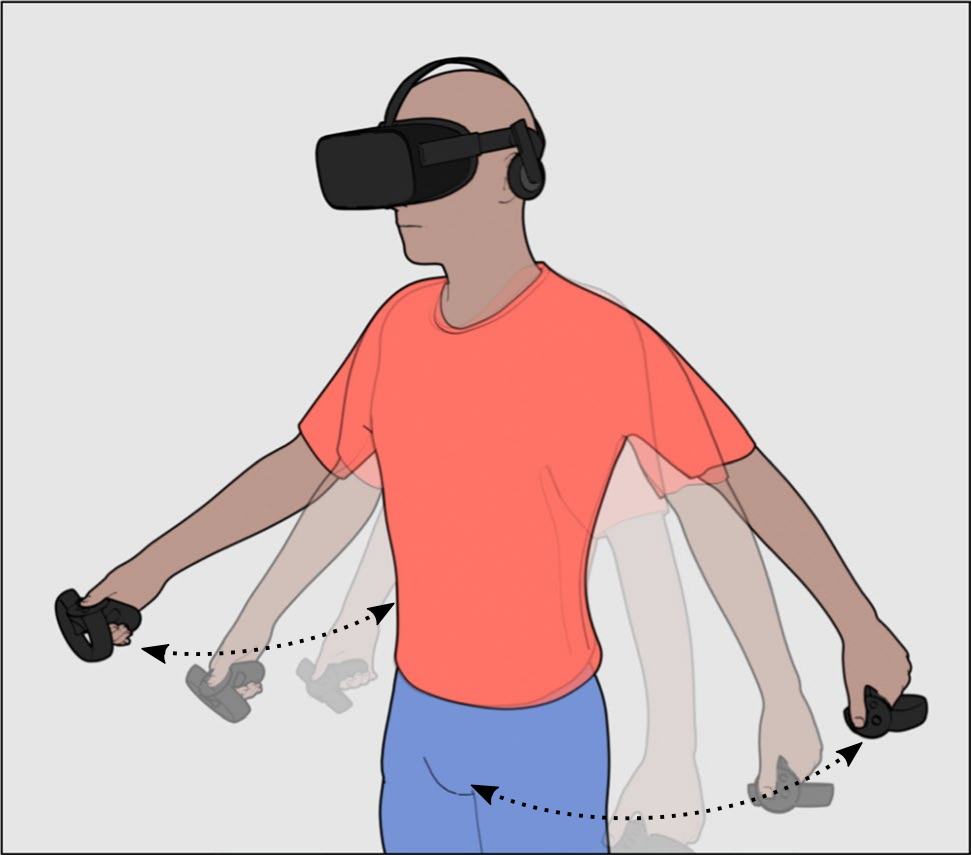
ArmSwinger allows users to walk or run in the virtual space by swinging their arms. The walking speed is determined by the swinging frequency, whereas the direction is controlled by hand rotation

There exist several locomotion methods (Boletsis 2017; Bozgeyikli et al. 2016; Nabiyouni et al. 2015) designed to allow users to walk in the virtual world, some of which are better than others at preventing motion sickness (McCauley and Sharkey 1992). The most common ones are natural walking, teleportation and stick walking: (1) natural walking is the best in terms of motion sickness and intuitiveness, but it requires that the space where users walk is large enough for the given application, and the VR system is untethered and portable; (2) teleportation is also good to limit motion sickness, but it is less intuitive than natural walking and sometimes not suitable for particular applications; (3) stick walking is the worst as far as motion sickness is concerned, because it creates a clear disconnection between what the vestibular system tells the user (that he/she is not moving) and what he/she sees. Harmonize requires that the VR system matches the AR system in terms of walking capabilities in large areas; however, to support the widest range of VR devices, it should be compatible with tethered systems such as the Oculus Rift. Moreover, teleportation was an unfeasible option to guarantee a similar experience. Since natural walking was not doable, Harmonize employs a version of stick walking based on an existing library named ArmSwinger.^5^ This locomotion method allows users to virtually walk by swinging their arms as if they were actually walking, as shown in Fig. 2.

### World state synchronisation and network model

The most common case of multi-user application is that of distributed model, where users are remotely connected and the computation is not centralised but distributed among multiple machines. In such a case, it is necessary to design the overall system so that it supports two basic mechanisms: (1) network communication among hosts and (2) application state synchronisation. Harmonize adopts a client–server network architecture to facilitate the synchronisation process: clients send inputs to the server, which processes them and then sends back the updated state of the world to each client. The advantages of this choice are multiple: (1) it is scalable with respect to the number of clients, because in order to have more clients it is required that only the server has more bandwidth; (2) some wearable devices may offer limited computational power, thus performing some of the application logic processing at the server side (e.g. for the physics simulation task) allows to unburden low-performance clients; (3) it is easier to implement a client–server architecture compared to a *peer-to-peer* one for applications that may involve more than two users.

**Fig. 3:**
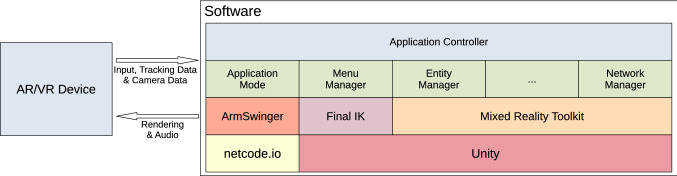
Arrows show the data-flow between the software layer and hardware devices

## Implementation

In order to support the widest set of devices and platforms, Harmonize is based on the popular Unity3D game engine. Unity3D is flexible and supports a large set of VR and AR devices and libraries, either natively or through third-party plugins. One of such plugins is the Mixed Reality Toolkit (MRTK), developed as an open-source project by Microsoft. The MRTK supports Windows Mixed Reality devices and OpenVR devices, such as Oculus Rift and HTC Vive. Moreover, the MRTK is highly modular and flexible, and each one of its modules can be replaced with a custom one. Figure 3 shows the software implementation of the proposed framework and how it is tied to Unity3D and MRTK, as well as other third-party libraries such as netcode.io and ArmSwinger, which will be discussed later on in Sects. 4.1 and 4.11.

### The communication protocol

In the context of networked solutions, two variables are particularly important: lag and jitter. The lag (or latency) is the time it takes for a packet to travel from the sender to the receiver; the jitter is the variation in the delay of received packets. Due to network congestion, improper queuing, or configuration errors, the delay between each packet can vary instead of remaining constant. In multi-user, interactive applications, especially if they involve AR and VR, visual imperfections and inconsistencies caused by both lag and jitter are easily recognised by the users (Beznosyk et al. 2011). Reliable transport protocols such as TCP adopt policies to guarantee the order and integrity of each exchanged packet, at the cost of introducing a perceivable delay in interactive applications. A fast, although unreliable, transmission protocol such as UDP is more suitable for this type of applications (Claypool et al. 2003; Ratti et al. 2010), and this is the reason why UDP is the favoured protocol in fast-paced multiplayer games such as First-Person Shooters (FPSs), and it has been chosen for Harmonize.

Unlike TCP, UDP is a connectionless protocol, so it lacks some basic functionalities, such as checking if a remote host is still reachable and can communicate with the local host. In order to have these functionalities in the system, the proposed framework uses a third-party library and protocol called netcode.io^6^, a connection-oriented protocol built on top of UDP and designed for high-performance and low-latency videogames.

### World synchronisation issues and solutions

**Fig. 4:**
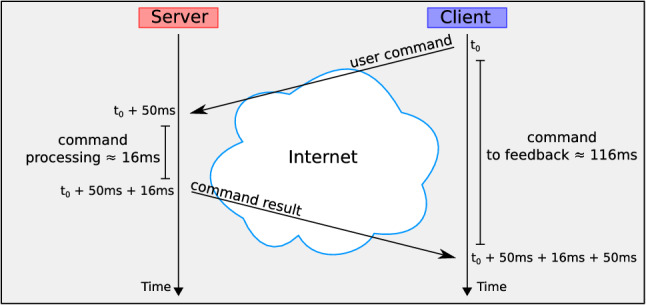
The network delay can be very high, thus negatively affecting the user experience. When a client sends a packet to the server (e.g. a user command), the server has to first receive it, then it can process it and finally it can send the result back to the client. A user might receive a feedback only after several milliseconds

Distributed architectures for multi-user applications introduce some issues caused not only by the network latency and jitter, but also by the distributed computation of the application logic. In order to mitigate the latency and to improve the perceived fluidity of the virtual scene, Harmonize uses a number of techniques which are typically used in existing fast-paced games. Figure 4 illustrates the negative effect of network delay on the data exchange among server and clients.

If the lag is greater than $\approx 50$ ms, not only users will start noticing it, but also the application may become unusable (Raaen and Kjellmo 2015). One of the techniques adopted to prevent this problem is called *client-side prediction*, and it consists in computing the result of the user’s input on the client instead of waiting for the server’s response. An example of this is the user using a virtual fire weapon; when they click the fire button, the weapon fires immediately, so the user can see and hear the shot.

Client-side prediction enables the user to perceive the application response as immediate, but the response computed by the server might differ from the one computed by the client. To solve this problem, each client corrects its state so that it is coherent to the one computed by the server that has authority over all the clients. This procedure is called *server reconciliation*. Client-side prediction and server reconciliation are implemented by separating each user command in primary and secondary effects (see Algorithm 1): primary effects are those effects that affect the state of the world, whereas secondary effects are just visual or sound effects that have no impact on the world, and only make the experience more interesting, usable and/or entertaining (e.g. the sound of a fire weapon).

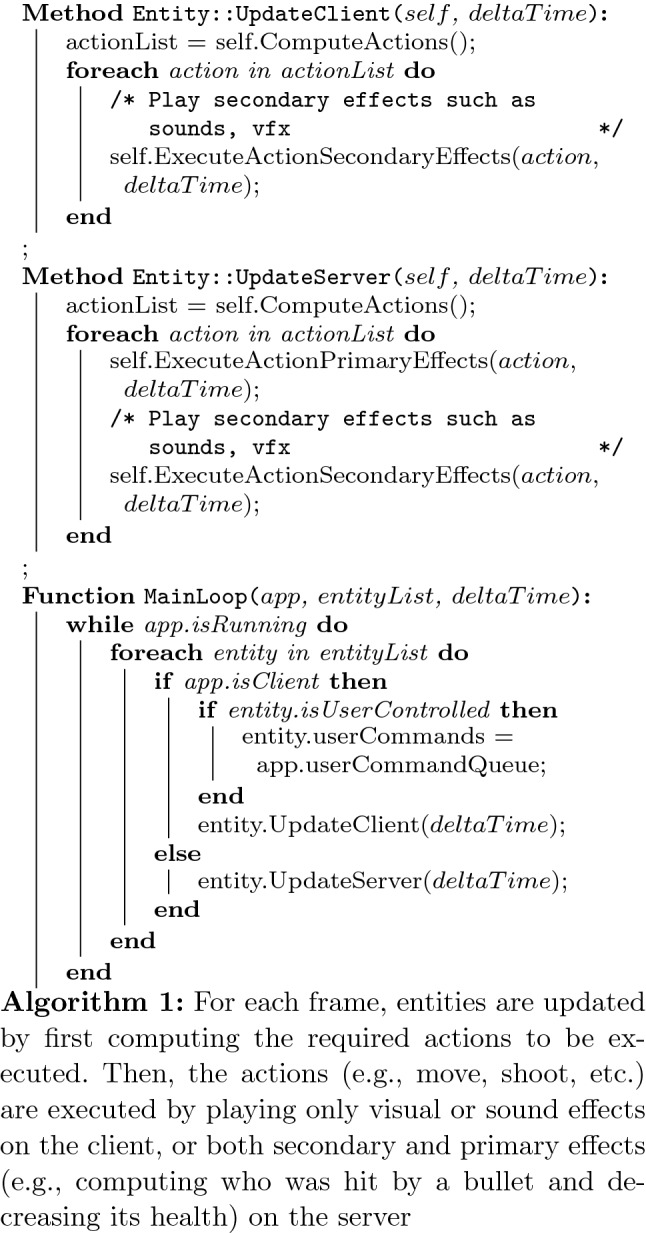



Another problem which may impact on the sense of fluidity is related to the methodology adopted by the server to process inputs: since it receives inputs from the clients at high frequency, instead of processing one command at a time, which would be CPU-demanding, the server batches the inputs in a buffer and processes them all at once at relatively low frequency (e.g. 10–20 times per second). This would add up to the network packet travel latency, resulting in an even greater perceived delay. This problem is addressed by allowing clients to use the past entity states in order to smoothly interpolate the position and orientation of each entity between the last received states. This technique is called *entity interpolation*.

With entity interpolation users would always see the past state instead of the current one, introducing logical inconsistencies, e.g. when two users interact on the same object at the same time. To solve this problem, since the server itself has the same information as the clients and can perform the same entity interpolation, it computes the results of user actions considering the status at the client side (e.g. what the user sees). As an example, this technique allows players in first person shooter videogames to continuously move and to be able to shoot each other.

### VR and AR in server-authoritative models

The server reconciliation technique previously described is a major concern when dealing with AR or VR technologies. In fact, let us consider what would happen if players penetrate a virtual wall with their heads and the server tries to control their positions. The server would determine that there is an obstacle, so it would push back the users accordingly. Since the users have physically moved their heads, they would feel the movement forward, but they would see the virtual scene move backwards, thus inducing motion sickness. In order to mitigate this problem, a mixed approach has been adopted: all the inputs related to the physical movement of the player are validated by the client, whereas all the other inputs are validated by the server. The VR client has to consider collisions of the user with the virtual environment, in order to avoid that virtual characters penetrate walls or other obstacles; this is implemented by taking advantage of the physics engine provided by Unity3D, which allows to define colliders which can be assigned to the world and to entities. Each client computes the new position and orientation independently for each frame, and then, it sends the updated user pose to the server, which broadcasts the user poses to all clients without changing them.

### Application controller and module classes

The client and the server are very similar in terms of modules, both rely on Unity3D and use the netcode.io library for network communications. Modules are C# class developed to handle different tasks. The module class implementation can be the same and shared across client and server, or there can be two different implementations for client and server. However, there are modules dedicated to solely the client or the server. The modularity allows easy adaptation and upgrade of the existing codebase with respect to different devices. The application controller has the role of coordinator, handling the modules and enabling them to exchange data.

### The network manager

This module, built on top of netcode.io (see Sect. 4.1), is addressed every time the system needs to establish a connection between a client and the server or one of them need to send or to receive a message. A dedicated thread manages both events and messages. The network manager provides dedicated methods to start or end a connection and to send messages. When required, the network manager can deal with message fragmentation. Moreover, since UDP is unreliable, the network manager extends it with a reliability layer for all those cases where it is strictly necessary.

### Virtual and real world alignment

In virtual reality, the user is tracked in the space relative to the external sensors, or relative to the starting position by sensors mounted on the VR device itself (Pinz et al. 2002). Since the user is immersed in a virtual world typically unrelated with the real place, this is sufficient to make the system work. However, AR devices such as the Microsoft HoloLens set the world centre in correspondence with the device position. Since it is not possible to predict nor it is advisable to enforce where the user will start the application, the scene containing the virtual objects will not be, most of the time, correctly aligned with the real world. Since Harmonize provides a shared environment for both AR and VR users, it is necessary to guarantee the consistency of the AR users’ position and rotation respect to the virtual world, despite the different coordinate systems. To solve this problem, anchors (Langlotz et al. 2011) have been used to memorise some absolute locations in the real-world environment. An anchor captures some colour or form features of a given location in order to recognise it later on when the device camera frames it again. For each anchor, its corresponding position and orientation is marked within the 3D virtual world. Once the AR device locates an anchor in the real world, it uses that as a reference point to compute a transformation that will be adopted to correctly align the detected anchor with its virtual location in the scene, effectively aligning the virtual world with the real play area. If more than one anchor is simultaneously located by the tracking device, the system uses the closest ones to compute the alignment transformation.

### The application mode

The application mode is a C# abstract class designed to allow application developers to define what goals the users have to pursue and what actions they can execute in the environment to reach those objectives. The mechanism to evaluate user (or rather entity) actions and update the application state is based on the message programming pattern: (1) at startup time, the application mode is set as listener for the entity messages; (2) when an entity executes a meaningful action, it sends a message to whichever is registered as listener; (3) the application mode receives the message and updates the state according to the previous state and the new input. Since the application mode is one of the modules used to determine the shared application state, this module is server-side only.

### Entities

Entities are human-controlled or computer-controlled actors and objects implementing specific functions and behaviours. In Harmonize an entity is both a class based on *MonoBehaviour* and a *prefab*; in Unity3D, MonoBehaviour is the base class for scripting, whereas prefabs are GameObjects with specific children and behaviours. The Entity class provides some methods which are called during the lifetime of the application, to allow developers to define its behaviour. Since the computation concerning the application rules is handled by the server, Harmonize provides some mechanisms to differentiate the logic among hosts, such as having some specific methods to be called solely from the server but not from the clients, and C# attributes to mark a specific method to be executed only by clients.

### World state synchronisation

The current state of an entity is determined by its variables (e.g. in videogames, a variable may store the health of the player), whereas the sequence of its executed methods represents its dynamics. In a shared application, it is required to synchronise both the entity state and the dynamics. The proposed framework implements a mechanism to automatically send the values of the class variables and the sequence of executed methods through the network. This mechanism is based on C# language *reflection* ability to introspect and examine a class structure. In order to synchronise entities across the server and clients, developers can use specially designed attributes to mark which variables have to be synchronised across all hosts. Moreover, in order to execute a method both locally and remotely, the framework provides an entity class method through which the method to be synchronised can be executed.

**Fig. 5:**
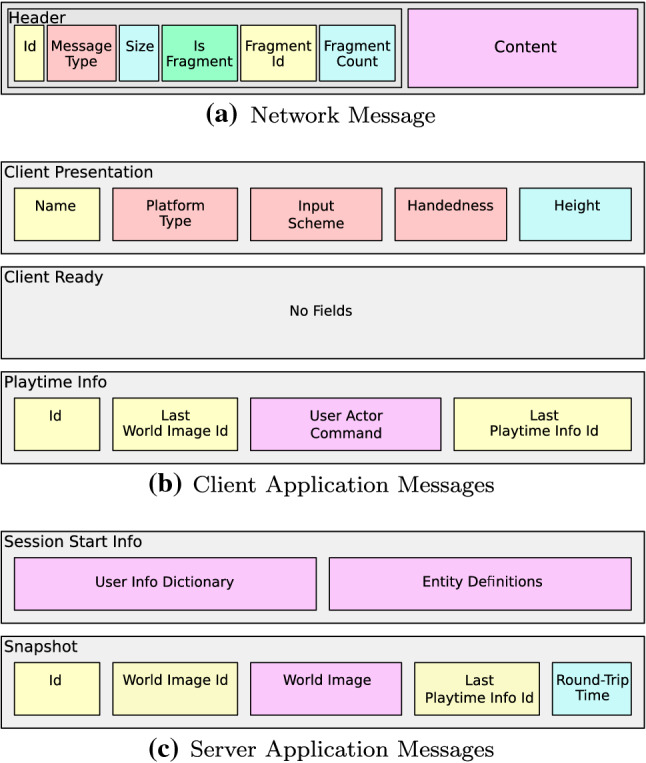
**a** The network message has some fields which allow to support reliability and fragmentation. **b** The *client presentation* message is sent from a client when it first connects to a server, whereas the *client ready* and *playtime info* messages are set just before a session starts and throughout the session, respectively. **c** The *start session info* message is sent by the server before a session starts and it contains the dictionary of the users that will participate in the session, whereas the *snapshot* is sent periodically throughout the session in order to update the world state on the clients’ side

Since the network communication is based on netcode.io, which in turn is based on the unreliable transport layer protocol UDP, there is no guarantee that messages will arrive in-order or, for that matter, will arrive at all. In order to circumvent this limitation without sacrificing the network communication speed, Harmonize implements a mechanism for no-data-loss communication by exploiting the fact that, unlike generic network solutions, an interactive VR/AR application expects that both the clients and the server exchange data at high frequency. Therefore, instead of sending a dedicated *ack* message to acknowledge the reception of a message, the proposed solution leverages the fact that both the server and clients continually send each other new data messages by including some information about the last received states and inputs. Since it is possible that the same world state or user inputs are received twice, the system is also able to discard already processed information. The structure of the network message and the main application messages sent by the clients and the server are shown in Fig. 5.

### Time synchronisation

Since for physical and technological reasons network communication cannot be instantaneous, interactive applications relying on the network infrastructure must include all necessary mechanisms to mitigate problems associated with communication delays. One of such problems is time synchronisation, which Harmonize solves by employing a straightforward mechanism: (1) periodically, each client receives a world state message, with a timestamp relative to the server clock; (2) each client computes the jitter as the deviation from the supposed periodicity (which is known because of the fixed communication period established at startup) and the real one, which is the difference between the instant it receives the message and the time it was supposed to arrive; (3) instead of updating the clock value with the one received by the server, each client computes the average over a fixed number of previously computed jitter values and uses this value to update its clock. In this way, the lag is averaged over multiple samples leading to less drastic visual spikes, while the system is still able to adapt to lag variability.

### ArmSwinger

ArmSwinger enables users to walk by swinging their arms. Users can control the direction and speed of walking by rotating their hands and by varying the swinging frequency. To prevent users from bumping against or penetrating virtual walls and other obstacles during walking, the proposed solution includes a method to detect obstacles in front of users and to automatically slow down the motion to a stop when needed. In order to reduce the chance of motion sickness, the walking speed of virtual characters has been limited to the walking speed of an average person. As shown in Sect. 7, nobody experienced motion sickness.

Since this library was initially implemented with HTC Vive and SteamVR 1.0 in mind, it binds directly to the inputs from the Vive Controllers. Since Harmonize does not utilise SteamVR, ArmSwinger was further developed to make it SDK-independent, by removing the references to SteamVR and modifying its public interface so that it is abstract and can be properly implemented for a given SDK with minimal code changes.

### Interaction methods

**Fig. 6:**
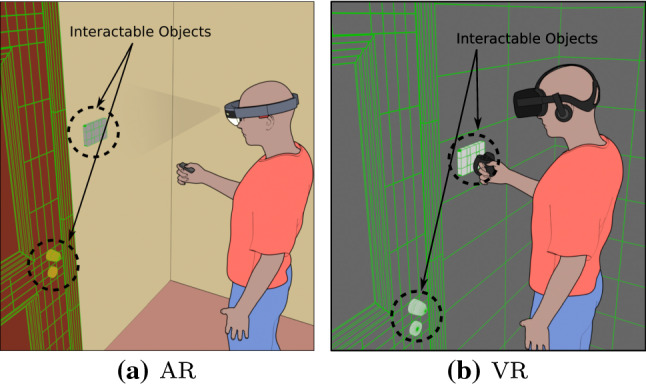
**a** The AR interaction method is based on gaze; the user looks at the object to be selected and then clicks the HoloLens clicker. **b** The VR interaction method is based on touch; the user has to virtually touch an object by moving their virtual hand close to the object, then they can click on a specific button in the Oculus Touch controller in order to interact with the object

Nowadays, VR systems use tracked controllers for interaction with the environment, even though recently some device producers have started experimenting with hand tracking. The most advanced industry-grade and consumer AR devices such as the Microsoft HoloLens have been using hand tracking and hand gesture recognition as a mean for interaction. The proposed framework implements a very common interaction metaphor requiring the user to virtually touch the objects to interact with them. Since having a motion controller per hand is very common in VR applications, Harmonize allows users to select their dominant hand at the start of the application. Hand gestures are automatically recognised by the HoloLens, and they are translated into input actions by the MRTK. Since the HoloLens 1 used for testing is not able to fully track the hands, but only recognise two basic gestures, Harmonize uses a metaphor requiring users to specify the desired action to be executed by looking at a specific class of object, then using the hand gesture to perform the action. VR systems also support the gaze-based interaction method described above. Figure 6 shows the interaction methods.

### AR and VR avatars

**Fig. 7:**
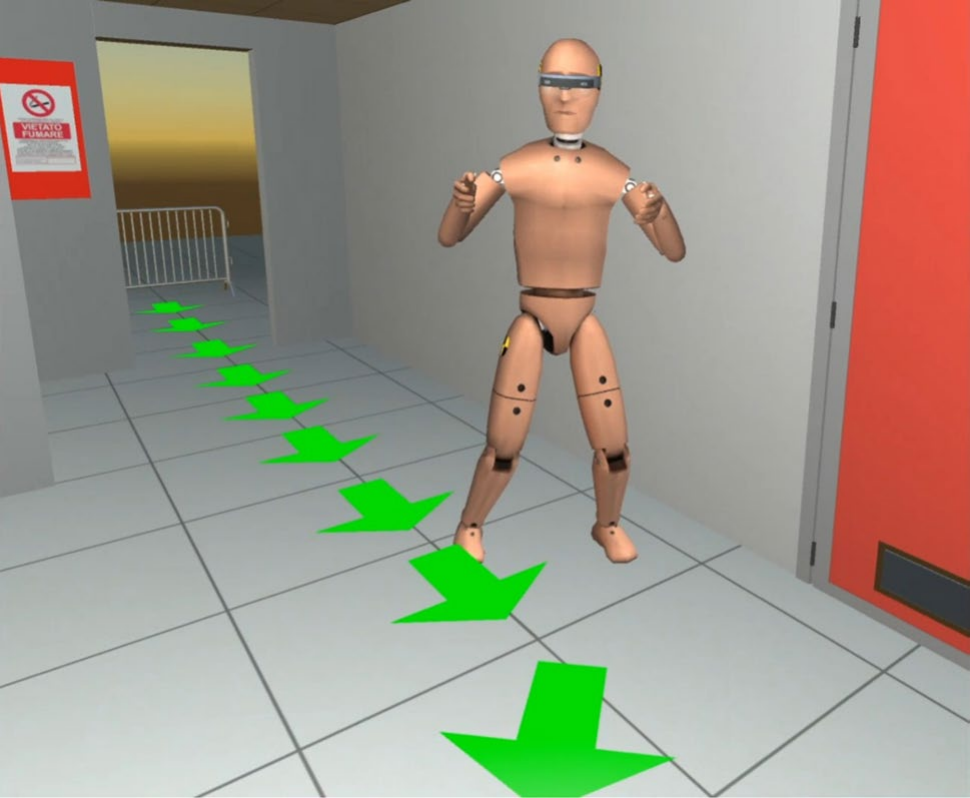
The avatar used to represent AR/VR users. The HoloLens model or the Oculus Rift model is added to the avatar based on the real device used

In order to increase the sense of presence and improve the application usability, Harmonize includes an existing third-party package for Unity3D developed by RootMotion and named Final IK. One of the scripts included in the package is aimed at VR systems and it is able to simulate the entire human animation skeleton using the tracked position and orientation of human limbs (e.g. the hands and the head) by means of *inverse kinematics*. Harmonize is able to load the IK avatar when needed; for example, AR users would not need to see an avatar of each other, since they can see the real bodies. Moreover, the proposed framework takes into account the height of the user, so that the avatar is correctly scaled to fit the real person. The user enters their height in the client application in the configuration stage, and then, this value is sent to the server in a presentation message (see Fig. 5b) in order to broadcast this information to all clients. The avatar model is then scaled in order to match the real user’s height. Figure 7 shows an avatar as seen by a VR user.

## The application lifetime

**Fig. 8:**
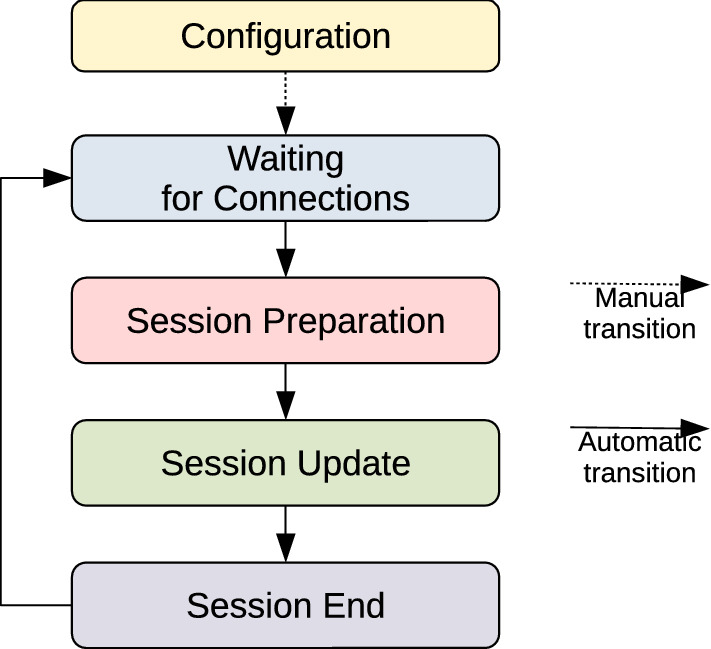
A simplified representation of the application state machine, showing the major states and transitions in the application lifetime

**Fig. 9:**
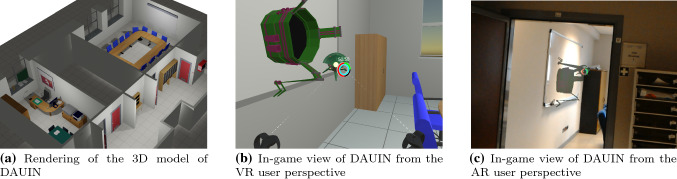
The area of the Department of Control and Computer Engineering (DAUIN) of Politecnico di Torino where the tests to evaluate Harmonize were conducted. **a** 3D model of DAUIN rendered by Blender; **b**,** c** same perspective of the conference room entrance as seen by the VR and AR users, respectively

### The server

In the starting state of the server, named *configuration*, the user is asked to specify some parameters: (1) the map where the play sessions will take place; (2) the network update frequencies for server-to-client and client-to-server message exchange; (3) the application mode, if the developers have included more than one mode for the given application (e.g. shooter games may have a *deathmatch* mode, a *capture the flag* mode, etc.). Once the configuration is confirmed, the server enters the *waiting for connections* state and it listens to connection requests by clients. When all the connected clients vote to start the play session, the server enters the *session preparation* state and it initialises all the objects’ data necessary for the given application mode and connected clients. Afterwards, the server enters the *session update* state, as it continually updates the application loop by processing the inputs received from clients. When the termination criteria for the given mode are met, the server enters the *session end* state and it sends termination messages containing the session results to all clients. After terminating the session, the server enters the *waiting for connections* state and it is again available to start a new session. The state machine representing the application lifetime is showed in Fig. 8.

### Clients

The client program can be executed on a VR or AR system, and it will automatically detect which one it is running on. At the start, users are asked to configure the client by inserting their height and dominant hand. Users are also able to select the desired interaction method among those available for the selected device. Once a user confirms the configuration, its client looks for an available server and it connects to it. When users are ready, they can vote to start the session, then the server sends them the starting message containing the initial state of the world. During the session, each client synchronises with the server while updating its own loop. When the session ends, users are shown the session results and they are able to start a new play session.

## A use case

The proposed system was tested with the Microsoft HoloLens 1 and the Facebook Oculus Rift CV1 with Touch Controllers. The HoloLens is a standalone AR HMD supporting SLAM-based tracking and hand gesture recognition for intuitive interactions with virtual objects. The Oculus Rift CV1 is a VR HMD supporting a camera-based 6DOF tracking in a small space around the external cameras. The Oculus Touch is the official motion controller for the Oculus Rift, and it is designed for sub-millimetre tracking of the user hands, thus allowing natural and intuitive interaction with the virtual environment.

The test system consisted of a desktop PC with Microsoft Windows 10 used both for the server and the VR client. The HoloLens acted as AR client, and a dedicated router was used to connect the server and the two clients in a local-area network using Wi-Fi. The test sessions were held in a dedicated area of the Department of Control and Computer Engineering of Politecnico di Torino, comprising a corridor, an office room and a meeting room. The test area was reconstructed in 3D using the Rhino^7^ and Blender^8^ software. (Figure 9a shows the rendered model. Figure 9b, c shows the VR and AR views, respectively.) The anchors for virtual world alignment were placed once before the tests took place. In order to ease the anchor placement process, anchors were placed in several wall corners of the play area.

An immersive game was developed with two modes: (1) in the *Deathmatch* mode players have to shoot and kill each other to gain points; (2) in the *Horde* mode players are expected to kill virtual enemies that are spawned in groups in predefined sectors of the play area. In order to make the experience richer and, therefore more interesting, some secondary tasks were added such as using virtual medical kits to recharge the avatar health level and ammunition to recharge the virtual firearm. Moreover, enemies are also able to shoot and they can move in random directions around the spawning point. With each new horde, enemies become faster and can travel greater distances. Since the Deathmatch mode is a subset of the Horde mode from a functional standpoint, only the latter mode was tested.

**Table 1:** The game experience core module outcomes

	Competence	Sensory	Flow	Tension	Challenge	Negative affect	Positive affect
AVG AR	2.31	2.625	2.23	0.65	1.69	0.362	3.06
SD AR	0.17	0.45	0.83	0.05	0.8	0.27	0.2
AVG VR	2.76	2.667	3.07	0.383	1.93	0.337	3.3
SD VR	0.04	0.32	0.49	0.38	1.11	0.28	0.1
Wilcoxon	0.042	0.527	0.042	0.102	0.593	1	0.042
Effect size	0.45	0.14	0.45	0.36	0.12	0	0.45

**Table 2:** The social presence module outcomes

	Empathy	Negative feelings	Behavioural involvement
AVG AR	1.475	0.84	1.47
SD AR	0.49	0.19	0.39
AVG VR	1.56	1.02	1.64
SD VR	0.64	0.45	0.37
Wilcoxon	0.248	0.223	0.027
Effect size	0.25	0.27	0.49

**Table 3:** The post-game module outcomes

	Positive Exp.	Negative Exp.	Tiredness	Returning to reality
AVG AR	1.92	0.29	0.25	0.67
SD AR	0.41	0.27	0.07	0.56
AVG VR	2.33	0.28	0.5	1.2
SD VR	0.44	0.31	0.14	0.69
Wilcoxon	0.026	1	0.18	0.109
Effect size	0.5	0	0.31	0.36

## Tests and results

Tests were aimed at evaluating different aspects of the proposed framework as a whole, including usability, engagement, graphical fidelity and fun. Since most of those aspects cannot be quantitatively measured, standard questionnaires such as the System Usability Scale (SUS) (Brooke 1996) and the Game Experience Questionnaire (GEQ) (IJsselsteijn et al. 2013) were used. The SUS is commonly used to measure, in a scale from 0 to 100, effectiveness, efficiency, simplicity and coherence of a given system. The GEQ tries to evaluate the game experience (fun, difficulty, involvement in the story), the social presence and post-game feelings (fatigue, shame or guilt). Quantitative data related to network and tracking system were also collected. Network data are useful because the AR system was connected to the server using Wi-Fi, while the VR system was directly tethered to it, resulting in less overall latency. On the other hand, the tracking data are useful because, contrarily to the Oculus Rift tracking system, which is quite reliable in proximity to the external sensors, the HoloLens tracking trades in robustness for large tracking volumes. Data were collected regarding both how many times the HoloLens suffered tracking loss and the delay to recover its position in space.

The tests involved 20 people in total (4 females and 16 males), who participated in groups of 2 people per test session. Most of the participants were knowledgeable about VR, AR or both. The age ranged from 19 to 30. Half of the users stated they use VR at least once per week, whereas the $20\%$ of users stated they use AR once per week. Before using the test system, users were explained how to use VR and AR devices, the objective of the immersive game they were going to play, and the extension of the playing area for AR players. After the first session, the participants were asked to fill in the section of the questionnaires pertaining the used system (AR or VR). Then, they switched devices for the second part of the test session and finally they completed the questionnaires.

Following IJsselsteijn et al. (2013), the GEQ outcomes have been clusterised in the game experience core module, social presence module and post-game module. As can be inferred from Table 1, the AR interface obtained lower scores than the VR one but it has been possible to detect statistically significant differences only for the competence, flow and positive affect sections. Referring to the social presence module (Table 2), both interfaces obtained relative low results and it has been possible to detect statistically significant differences only for the behavioural involvement category. Finally, the post-game module outcomes show that the VR interface generally obtained higher scores with respect to the AR one (Table 3). Specifically, it seems the users spent a much more positive experience with the VR interface than the AR one. The result is also confirmed by the post hoc test that shows statistically significant differences.

Referring to the statistically significant outcomes of Tables 1, 2 and 3, the post hoc analysis shows small effect sizes *d* for the related categories (the effect size is a measure of the “strength” of the differences among the average values (Cohen 2013)), thus suggesting that the VR and AR interfaces do not provide substantially different experiences.^9^

Overall, the experience was positively received by the participants for both VR and AR, although the social presence score was significantly lower than the other scores. This is believed to be due to at least three reasons: 1) the users could not communicate during the play session, 2) the virtual scene was not realistic enough and 3) the user avatars were not realistic. All these issues will be examined in order to evaluate their impact on the social presence in future work.

In order to improve the user engagement, the game should be designed to allow a tighter cooperation among players, for example by including ways to combine users abilities to execute new attack or defence moves that cannot be executed by a single player. The post-game score could be improved by considering breaks during the game sessions, e.g. by designing the game modes so that to alternate frenetic moments with a lot of action and more relaxed times.

The participants were also asked to optionally remark which aspects they considered improvable. The most frequent remarks were: (1) lower field of view of the HoloLens compared to the Oculus Rift, making it hard to see the virtual objects, (2) a less intuitive gaze-based targeting system for the AR user compared to the more natural VR system gun-style, (3) inability to communicate by voice, (4) virtual signs and indicators not always well visible.

**Fig. 10:**
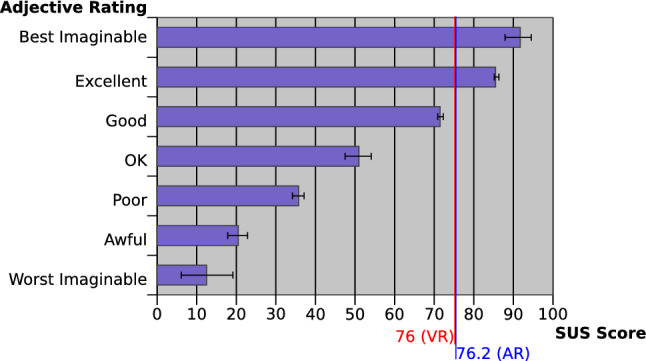
Correlation between the SUS score and adjective ratings (Bangor et al. 2009). The score of the proposed system is shown in figure separately for VR and AR

Concerning the SUS results (Fig. 10), the AR and VR interfaces obtained a similar positive score and they were both considered equally suitable to interact in the proposed environment (the Wilcoxon signed rank test showed a $p=1$ with effect size $d=0$).

Regarding the quantitative data, measurements showed that the HoloLens would lose tracking approximately 1.35 times per session, and that it was able to recover from that in $5 \pm 2.3$ s. The measured network round-trip time of about 200 ms and the packet loss of $1\%$ did not affect the experience, as respondents did not remark any issue related to that.

## Conclusions and future works

This paper presents Harmonize, a novel framework to deploy applications based on shared environments for VR and AR users. The most relevant novelties of the proposed framework are two: 1) the framework enables developers to create shared environments offering a similar experience to both VR and AR users in a multi-user context; 2) the framework is hardware-independent and it has been designed to be extendable to novel hardware. The proposed framework has been tested on a use case based on see-through AR displays and VR head-mounted displays (HMD) with motion controllers. In order to test and to evaluate the validity of Harmonize, an immersive game has been implemented. The assessment of the system by the System Usability Scale (SUS) questionnaire and the Game Experience Questionnaire (GEQ) shows a positive evaluation towards the proposed framework. Despite that the VR interface has been generally preferred by the users, only few statistically significant outcomes have been detect with small effect sizes; thus, it is not possible to conclude that the users spent different experiences using the AR and VR devices. Future works will be focused on investigating novel interaction paradigms allowing users immersed in different reality of the reality–virtuality continuum to collaborate together. The Harmonize framework will be useful to further research how users in one reality can visualise or be made aware of what is happening in other realities, how users can express interaction intents that originate in one reality but affect another and how to evaluate the feeling of social presence across realities. Other research topics strictly related to the Harmonize framework include: supporting other paradigms for recreating the virtual environment, such as fast 3D reconstruction by deep learning classification algorithms; investigating the creation of novel user interfaces to overcome the hardware limitation of current AR glasses (limited field of view and low contrast); researching novel interaction metaphors to guarantee homogeneous experiences despite diversity in input devices.

## Footnotes

1 https://www.oculus.com/.

2 https://www.vive.com/eu/.

3 Unity3D can be used free of charge for non-commercial academic research.

4 http://www.openscenegraph.org/.

5 https://github.com/ElectricNightOwl/ArmSwinger.

6 https://github.com/networkprotocol/netcode.io.

7 https://www.rhino3d.com.

8 https://www.blender.org.

9 The effect sizes have been computed as $d=\frac{Z}{\sqrt{N}}$, refer to (Tomczak and Tomczak 2014).
